# Polymerase chain reaction and histology in diagnosis of placental malaria in an area of unstable malaria transmission in Central Sudan

**DOI:** 10.1186/1746-1596-6-128

**Published:** 2011-12-23

**Authors:** Haggar M Elbashir, Magdi M Salih, Elhassan M Elhassan, Ahmed A Mohmmed, Mustafa I Elbashir, Ishag Adam

**Affiliations:** 1 Faculty of Medicine, University of Khartoum, P.O. Box 102, Khartoum, Sudan; 2 Faculty of Medical Laboratory Sciences, University of Khartoum, P.O. Box 302, Khartoum, Sudan; 3 Faculty of Medicine, University of Gezira, P.O. Box 816, Medani, Sudan; 4 Faculty of Medicine, The National Ribat University, P.O. Box 1157, Khartoum, Sudan

## Abstract

**Background:**

Prevalence of placental malaria has been widely used as a standard indicator to characterize malaria infection in epidemiologic surveys. Placental malaria poses a greater diagnostic challenge, accurate and sensitive diagnostic tool for malaria infections in pregnancy is needed.

**Methods:**

A cross sectional study was conducted at Medani Hospital, which serves catchment area which is characterized by unstable malaria transmission. One hundred and seven placentae were investigated for malaria infection using polymerase chain reaction (PCR) and histology.

**Results:**

out of 107 investigated placentae, 33 (30.8%) and 34 (31.8%) were positive for malaria by histology (two (2%) and 31(29.0%) were acute and past infections, respectively) and PCR, respectively. Out of 33 positive by histology, 15 were positive by the PCR while 18 were negative. The sensitivity of the PCR was 45.5% (95% CI: 29.2%- 62.5%). Out of 74 which were negative by histology, 19 were positive by the PCR. This is translated in specificity of 74.3% (95% CI: 63.5%- 83.3%). Of those tested positive by the PCR, 15 were positive by the histology, while 19 were negative. This is translated into a positive predictive value of 44.1% (95% CI: 28.3%- 61.0%). Of those 73 tested negative by the PCR, 55 were negative according to histology while 23 were positive. This is translated into a negative predictive value of 75.3% (95% CI: 64.5%-84.2%).

**Conclusion:**

PCR had low sensitivity and specificity in comparison to placental histology, perhaps because the vast majority of the placental infections were past infections. Further research is needed.

## Introduction

Malaria during pregnancy is a major public health problem in tropical and subtropical regions of the world [1]. It has been estimated that, of 85.3 million pregnancies in areas with *Plasmodium falciparum *transmission, 54.7 million occurred in areas with stable transmission and 30.6 million in areas with unstable transmission [2]. In Sudan, malaria during pregnancy is a major health problem where pregnant women are more susceptible to malaria regardless to their age or parity [3-5]. Malaria has serious adverse effects on pregnancy and it is a leading cause of maternal and perinatal mortality in Sudan [6-8].

During pregnancy, adhesion of *P. falciparum*-infected erythrocytes to syncytiotrophoblast leads to parasite sequestration in the intervillous space. The parasite adheres specifically to chondroitin sulfate-A expressed on syncytiotrophoblast [9]. Therefore placental malaria infection may be detected in the absence of peripheral blood parasitemia [5,10]. Placental malaria infection is widely recognized as indicator for malaria infection in epidemiologic surveys for both operational and research purpose [11]. Malaria during pregnancy poses a greater diagnostic challenge especially in area of unstable malaria transmission where, the rates of placental malaria blood film microscopy have higher than that of the peripheral blood film microscopy [12,13]. The placental histology is the 'gold standard' of malaria diagnosis during pregnancy for epidemiological or study purposes, it can show different grade of infections e.g. active infection, past or chronic infection [13,14]. It is, however, frequently not available in most settings such as sub-Saharan Africa, relatively costly and labour intensive [12]. Polymerase chain reaction (PCR) is the other alternative and is widely used for malaria infection during pregnancy [15,16]. There are few published data on placental malaria infections that comparing PCR with placental histology [16,17]. The current study was conducted at Medani Maternity Hospital which is located in an area characterized by unstable malaria [18], to investigate diagnostic performance of PCR in comparison with that of the placental histology so as to add on the previous studies on the diagnosis of malaria during pregnancy in the area [19].

## Methods

A cross sectional study was conducted during the period of October 2010, at the labour ward of Medani Maternity Hospital, Central Sudan. The area is characterized by unstable malaria transmission and *P. falciparum *is the sole malaria species in the area [18]. Medani Maternity Hospital has a tertiary care for women who receive antenatal care at the hospital as well as for referrals from the other clinics and hospitals, and women who are close to the hospital facility. All women with risk factors or obstetric complications are referred to the hospital. The referral criteria are not strictly adhered to and many patients without any significant complications deliver at the hospital.

### Giemsa stained light microscopy

After signing an informed consent, placental blood films were prepared, the slides were Giemsa-stained and the number of asexual *P. falciparum *parasites per 200 white blood cells was counted and double-checked blindly by an expert microscopist.

### Placental histology

**Figure 1:**
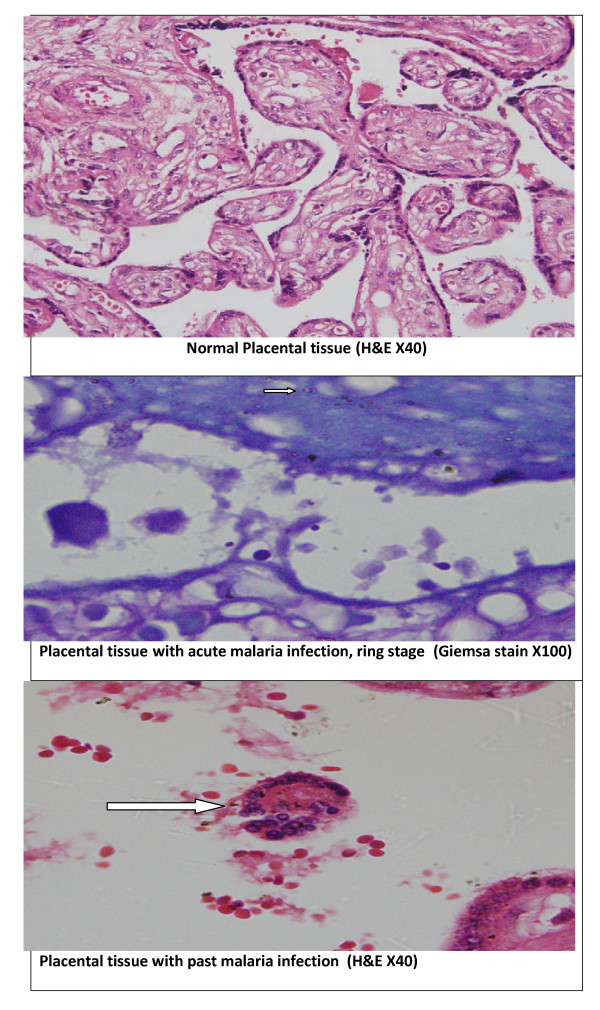
**Placental histology and malaria infections**.

Full thickness placental blocks of around 2-3 cm were taken from the placenta, kept in neutral buffer formalin for histopathology examinations. Placental malaria infections were characterized based on the classification of Bulmer *et al *[14]: uninfected (no parasites or pigment), acute (parasites in intervillous spaces), chronic (parasites in maternal erythrocytes and pigment in fibrin or cells within fibrin and/or chorionic villous syncytiotrophoblast or stroma), past (no parasites and pigment confined to fibrin or cells within fibrin), Figure 1. Because the samples were fixed in buffered formalin, formalin pigment formation, which has similar optical characteristics and polarized light activity to malaria pigment was not detected [20].

### Parasite DNA Extraction and PCR

*P. falciparum *DNA extraction and PCR assays were performed as described in details in our previous work [15]. In summary, three drops of blood were collected on to a piece of filter paper, from the maternal side of each placenta. Blood samples were air-dried and stored at ambient temperature, in individual sterile plastic bags. The dried specimens were later transported for processing and analysis in Khartoum. Approximately 25 μl (corresponding to approximately 1/3 of the spot) of blood was punched out from the dried blood spots. This piece was then washed with distilled water and directly placed in a PCR reaction tube of 25 μl in all PCR reactions. A negative control sample with no template DNA and an internal positive control were used for quality control. Genomic DNA was checked, in an assay based on a nested PCR, for DNA from *P. falciparum *[21]. These PCR were performed by two of our team (HMI and MIE) and who were blinded about the histological results.

### Ethics

The study received ethical clearance from the Research Board at the Faculty of Medicine, University of Khartoum.

### Statistics

Data were entered in computer using SPSS (statistical package for social sciences) for soft ware version 19.0 for analysis. Proportions were compared using X^2 ^test. Sensitivity, specificity, positive predictive value and negative predictive value were calculated. Sensitivity of the PCR was calculated as true positives/(true positive + false negatives), specificity as true negatives/(true negatives + false positives), positive predictive value as true positives/(true positives + false positives), negative predictive value as true negatives/(true negatives + false negatives) [22].

## Results

One hundred and seven placentae were investigated by histology and PCR. The mean (SD) age of the parturient women was 26.4(6.9) years. Thirty (28.0%) of these women were primigravidae. None of the women used intermittent preventive treatment (IPT). The microscopy showed that only one (0.9%) placental blood film was positive for *P. falciparum *malaria.

The histology and PCR showed that 33 (30.8%) and 34 (31.8%) of these 107 placentae were positive for malaria. There was no significant difference in the results of the histology [11/30 (33.3%) vs. 22/77 (28.6%), p = 0.4] and PCR [12/30 (40.0%) vs. 22/77(28.6%), p = 01] when primigravidae were compared to multigravidae women. Two (2%) were acute infection, while 31(29.0%) were past infection by histology. Out of 33 positive tested by histopathology, 15 have been found to be positive by the PCR while 18 were negative, Table 1. Both the two chronic placental infections were positive by PCR. Thus, the sensitivity of the PCR was 45.5% (95% CI: 29.2%- 62.5%). Out of 74 which were negative by histology, 19 were positive by the PCR. This is translated in specificity of 74.3% (95% CI: 63.5%- 83.3%), Table 2. Of those tested positive by the PCR, 15 were positive by the histology, while 19 were negative. This is translated into a positive predictive value of 44.1% (95% CI: 28.3%- 61.0%). Of those 73 tested negative by the PCR, 55 were negative according to histology while 23 were positive. This is translated into a negative predictive value of 75.3% (95% CI: 64.5%-84.2%), Table 2.

**Table 1:** Polymerase chain reaction results compared to placental histology

		**PCR**		**Total**
		**Negative**	**Positive**	
			
Placental histology	Neg	55	19	74
	Post	18	15	33
Total		73	34	107

**Table 2:** Diagnostic performance of PCR using placental histology as gold standard

Performance	% (95% Confidence Interval)
Sensitivity	45.5% (29.2%- 62.5%)
Specificity	74.3% (63.5%- 83.3%)
Positive predictive value	44.1% (28.3%- 61.0%)
Negative predictive value	75.3% (64.5%-84.2%)

## Discussion

The main findings of the current study were; the prevalence of placental malaria infections was 0.9%, 30.8% and 31.8% by microscopy, histology and PCR, respectively, the sensitivity and specificity of the PCR was 45.5% and 74.3%, respectively compared with placental histology. Recently, two studies in the Eastern Sudan have shown that, the prevalence of placental *P. falciparum *was 1.7% (5/293) and 3% (7/237) by microscopy examinations and the prevalence was 32.0% and 19.5% by the histology [5,23]. Interestingly, 40 (32%) of the 125 smear-negative pregnant women in the Eastern Sudan had submicroscopic *P. falciparum *(PCR) infections [15]. The low sensitivity and specificity of PCR in this study goes with the recent previous observation from Colombia where the sensitivity and specificity of PCR (histology was the gold standard) was 47% and 77%, respectively [17]. However, our findings should be compared with the results of later study cautiously, because the vast majority of the infections in our study were past infection while one third (33%) of the infections in the later one were acute and the rest were chronic (7%) and past (60%) infections.

Histological examination of the placenta biopsy is considered the gold standard for diagnosis of placental malaria. Furthermore, the placental histology indicates the presence of malaria parasites and pigment in the placental tissue. Therefore malaria infection can be classified in active (parasites in the placenta), chronic (parasites and pigment in placenta), past (only pigment in placenta) and no infection (no parasites or pigment in placenta). However, due to limited resources, technical expertise, such placental histology is rarely available in endemic areas with low recourses [12-14]. PCR is the most sensitive tool for detection parasites (for both peripheral and placental malaria), however, requires a trained staff and specialized equipment, which might not available in the field in settings with low resources [13,16]. Furthermore PCR-based methods have no standard in method for DNA extraction, choice of primer sets, and amplification protocol [24]. On the other hand PCR is very sensitive in detecting *Plasmodium *nucleic acids, yet it is not obvious if this is derived from a non-viable sequestered parasite, or a viable one or even gametocyte. In the current study the different rates of PCR-positivity reported between women with past infections and normal placentae is a significant finding. PCR in the women with past infections may detect non-viable parasites in treated women, viable sub microscopic persisting infections or re-infections in women at increased risk for infection. It is worth to be mentioned that, although placental histology can provide a quantitative result based on the number of parasites, conventional PCR (not the real time PCR) gives a qualitative result but allow species discrimination [25].

## Conclusion

PCR had low sensitivity and specificity in comparison to placental histology, perhaps because the vast majority of the placental infections were past infections. Further research is needed.

## Competing interests

The authors declare that they have no competing interests.

## Authors' contributions

HME and IA designed the study, EMH conducted the clinical work. MMS, AHM, HME and MIE conducted the lab work. IA and EME participated in the statistical analyses. All the authors approved the draft and the final paper.
